# Supplementary material for: Identification and Functional Characterization of WRKY, PHD and MYB Three Salt Stress Responsive Gene Families in Mungbean (Vigna radiata L.)
Source: Genes (Basel). 2023 Feb 10;14(2):463. doi: 10.3390/genes14020463 (PMC9956968; doi:10.3390/genes14020463)
Supplement: Supplementary file 1 [file genes-14-00463-s001.zip › genes-2119742-supplementary.pdf]

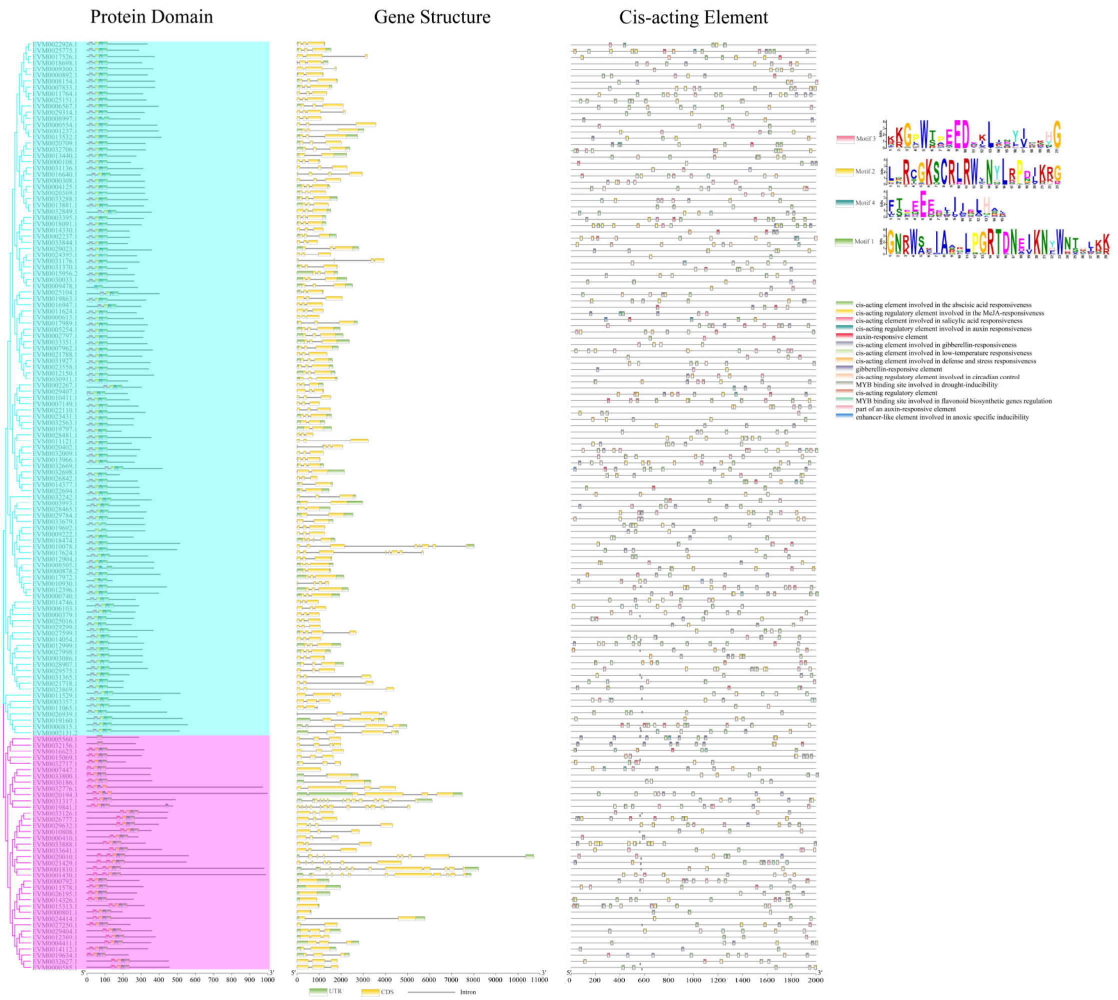

Supplementary Figure S1. Protein domain, gene structure, cis-acting element analysis of *VrMYBs*.

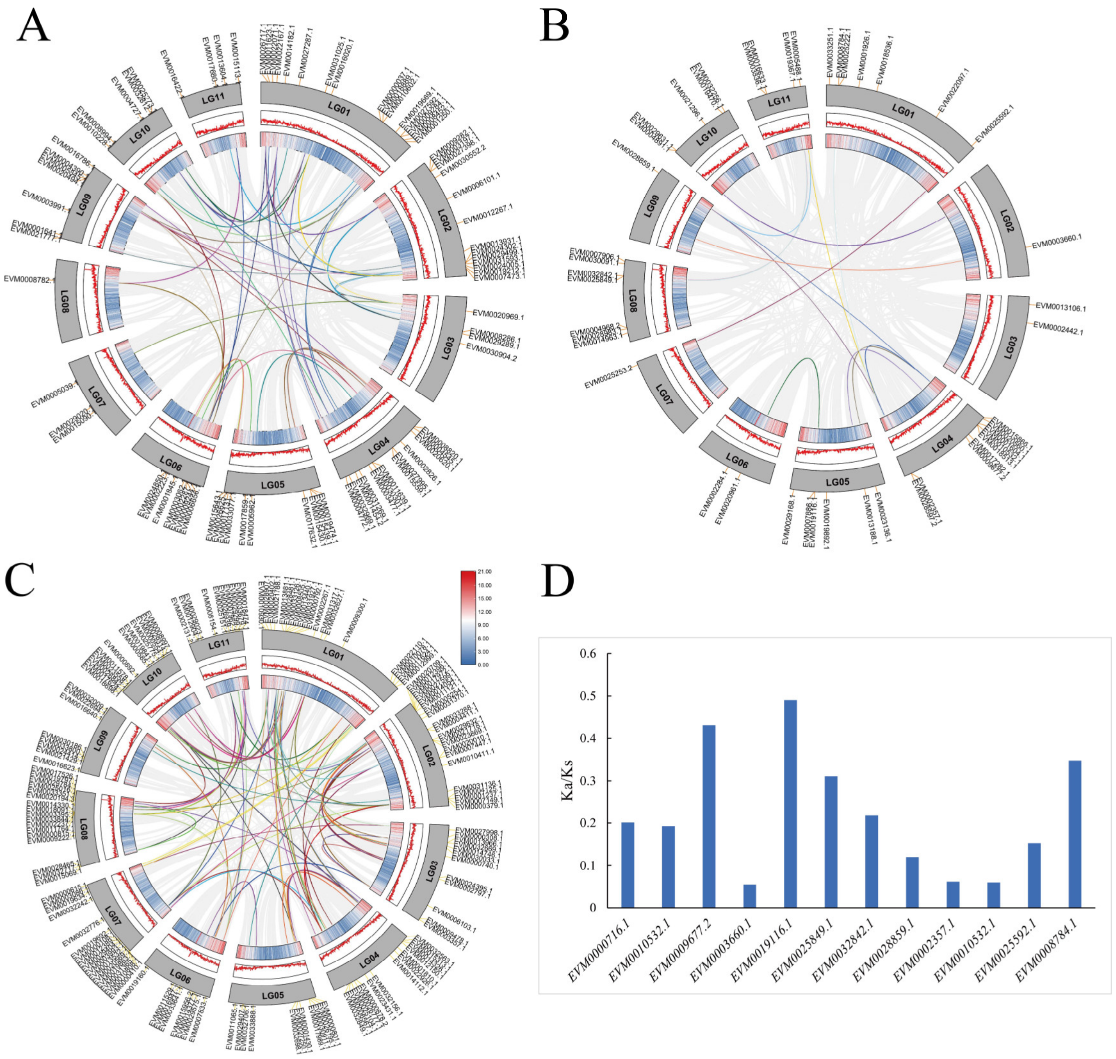

Supplementary Figure S2. Synteny analysis and calculation of Ka/Ks value. **A, B, C** collinearity analysis diagram of VrWRKYs, VrPHDs and VrMYBs. **D**, Ratio of heteronymous substitution (Ka) to synonymy substitution (Ks).

Supplementary Table S1. The list of primers used in the study.

Primer	Sequence (5'to3')	Purpose	Products(bp)
EVM0010532-F	ATGGAACCAAAGGATGAGAG	Quantitative Real time RT-qPCR	179
EVM0010532-R	TGAGCCTTCGCAGGAGTAAT	Quantitative Real time RT-qPCR	
EVM0027733-F	GACAAGGACCGAGGATACTGA	Quantitative Real time RT-qPCR	190
EVM0027733-R	TCCATTCACTTCCATTCTCC	Quantitative Real time RT-qPCR	
EVM0015430-F	AAGGGTCAAGTCCAGTTTCT	Quantitative Real time RT-qPCR	174
EVM0015430-R	CCTGAAATGGCAGTGGCAAG	Quantitative Real time RT-qPCR	
EVM0019669-F	AGGAGACAGAAGGGTGTAGGCT	Quantitative Real time RT-qPCR	278
EVM0019669-R	TGGTTGTGGTCGGAAGAGTAAGT	Quantitative Real time RT-qPCR	
EVM0027995-F	TTCAAGACCAGGTTGCTCAG	Quantitative Real time RT-qPCR	137
EVM0027995-R	GGAACCTCTGCGATCCTTTG	Quantitative Real time RT-qPCR	
EVM0000108-F	TTGGTAACAGGTGGTCTAAG	Quantitative Real time RT-qPCR	266
EVM0000108-R	ATCAGGGTCAAAGGGTCCAA	Quantitative Real time RT-qPCR	
EVM0009300-F	GGAACAAACACCCTCCAAGT	Quantitative Real time RT-qPCR	110
EVM0009300-R	GAATAGTCACTGGGCGTTTG	Quantitative Real time RT-qPCR	
VrACTIN3-F	TTCTTTATGGTTGGGTTTGC	Quantitative Real time RT-qPCR	192
VrACTIN3-R	GCTCGTCTACCTCCTTTGTG	Quantitative Real time RT-qPCR	

Supplementary Table S2. VrWRKY gene family members and the physical length of their encoded proteins.

Gene name	Sequenced gene ID from 'Sulv No. 1'	Chromosome	Coding sequence length/bp	5′ upstream regions	Amino acid length
VrWRKY1	EVM0026717	LG01	1206	3112176	402
VrWRKY2	EVM0011623	LG01	1053	3781516	351
VrWRKY3	EVM0022071	LG01	978	5156801	326
VrWRKY4	EVM0022167	LG01	1512	6945671	504
VrWRKY5	EVM0014182	LG01	1767	10536372	589
VrWRKY6	EVM0027287	LG01	966	16776664	322
VrWRKY7	EVM0031025	LG01	1455	28369389	485
VrWRKY8	EVM0016020	LG01	1119	31291387	373
VrWRKY9	EVM0000037	LG01	1089	55671207	363
VrWRKY10	EVM0018889	LG01	1296	56687234	432
VrWRKY11	EVM0010602	LG01	1473	56840132	491
VrWRKY12	EVM0019669	LG01	783	63843482	261
VrWRKY13	EVM0027592	LG01	570	66573671	190
VrWRKY14	EVM0009284	LG01	699	66698689	233
VrWRKY15	EVM0008452	LG01	393	68813773	131
VrWRKY16	EVM0001250	LG01	1086	69839198	362
VrWRKY17	EVM0009282	LG02	1590	4147587	530
VrWRKY18	EVM0003197	LG02	1248	4484969	416
VrWRKY19	EVM0027398	LG02	777	4560357	259
VrWRKY20	EVM0030552	LG02	1482	8046337	494
VrWRKY21	EVM0006101	LG02	579	18629113	193
VrWRKY22	EVM0012267	LG02	897	30202633	299
VrWRKY23	EVM0013931	LG02	1602	42634299	534
VrWRKY24	EVM0024302	LG02	1041	47543054	347
VrWRKY25	EVM0029499	LG02	552	47564500	184
VrWRKY26	EVM0031593	LG02	2097	47967250	699
VrWRKY27	EVM0024005	LG02	639	48687308	213
VrWRKY28	EVM0019212	LG02	1581	49351573	527
VrWRKY29	EVM0007473	LG02	1557	50006183	519
VrWRKY30	EVM0020969	LG03	966	6137107	322
VrWRKY31	EVM0008286	LG03	1365	15313098	455
VrWRKY32	EVM0029289	LG03	960	16027392	320
VrWRKY33	EVM0030904	LG03	1197	20214750	399
VrWRKY34	EVM0008820	LG04	531	4266461	177
VrWRKY35	EVM0004545	LG04	834	4867063	278
VrWRKY36	EVM0020620	LG04	891	6445015	297
VrWRKY37	EVM0002826	LG04	1047	12891450	349
VrWRKY38	EVM0027995	LG04	861	17360828	287
VrWRKY39	EVM0015559	LG04	957	17371935	319

VrWRKY40	EVM0011639	LG04	591	28343990	197
VrWRKY41	EVM0030301	LG04	720	28449806	240
VrWRKY42	EVM0003477	LG04	435	30266261	145
VrWRKY43	EVM0031269	LG04	1650	35028223	550
VrWRKY44	EVM0001454	LG04	1473	36804500	491
VrWRKY45	EVM0032969	LG04	1332	39963868	444
VrWRKY46	EVM0004772	LG04	888	41357261	296
VrWRKY47	EVM0019474	LG05	492	4369923	164
VrWRKY48	EVM0015439	LG05	807	4637516	269
VrWRKY49	EVM0015430	LG05	783	4658920	261
VrWRKY50	EVM0017632	LG05	987	7750570	329
VrWRKY51	EVM0005962	LG05	621	30094066	207
VrWRKY52	EVM0017859	LG05	1722	32989227	574
VrWRKY53	EVM0033077	LG05	978	39940211	326
VrWRKY54	EVM0017131	LG05	1284	40535939	428
VrWRKY55	EVM0026625	LG05	1755	40978339	585
VrWRKY56	EVM0015643	LG05	1521	41719441	507
VrWRKY57	EVM0006656	LG06	1710	5689023	570
VrWRKY58	EVM0009244	LG06	1494	7722797	498
VrWRKY59	EVM0002287	LG06	1065	8084226	355
VrWRKY60	EVM0003062	LG06	1116	8781604	372
VrWRKY61	EVM0001845	LG06	1581	12954236	527
VrWRKY62	EVM0002223	LG06	1749	16736348	583
VrWRKY63	EVM0024850	LG06	1416	17673535	472
VrWRKY64	EVM0015090	LG07	477	13980944	159
VrWRKY65	EVM0029020	LG07	660	16339447	220
VrWRKY66	EVM0005039	LG07	1194	28636478	398
VrWRKY67	EVM0008782	LG08	1770	27359799	590
VrWRKY68	EVM0021777	LG09	798	359586	266
VrWRKY69	EVM0001641	LG09	678	1432518	226
VrWRKY70	EVM0003991	LG09	1476	12551088	492
VrWRKY71	EVM0020494	LG09	957	28355438	319
VrWRKY72	EVM0005845	LG09	618	28541050	206
VrWRKY73	EVM0004390	LG09	1083	29463818	361
VrWRKY74	EVM0016786	LG09	903	34297112	301
VrWRKY75	EVM0010228	LG10	1044	1558931	348
VrWRKY76	EVM0008994	LG10	954	4292483	318
VrWRKY77	EVM0004727	LG10	1440	20199581	480
VrWRKY78	EVM0032812	LG10	879	24666454	293
VrWRKY79	EVM0025273	LG10	1371	25044254	457
VrWRKY80	EVM0016422	LG11	2241	617681	747
VrWRKY81	EVM0017680	LG11	768	16131987	256
VrWRKY82	EVM0013604	LG11	1845	20050310	615
VrWRKY83	EVM0015113	LG11	918	26029672	306

Supplementary Table S3. VrPHD gene family members and the physical length of their encoded proteins.

Gene name	Sequenced gene ID from 'Sulv No. 1'	Chromosome	Coding sequence length/bp	5′ upstream regions	Amino acid length
VrPHD1	EVM0033251	LG01	762	1393887	254
VrPHD2	EVM0008784	LG01	2097	5382545	699
VrPHD3	EVM0025222	LG01	2214	5705718	738
VrPHD4	EVM0001926	LG01	3708	14335905	1236
VrPHD5	EVM0018536	LG01	2610	22501597	870
VrPHD6	EVM0022097	LG01	2418	51222160	806
VrPHD7	EVM0025592	LG01	2166	71341992	722
VrPHD8	EVM0003660	LG02	723	40650417	241
VrPHD9	EVM0013106	LG03	2772	3408311	924
VrPHD10	EVM0002442	LG03	2139	11584544	713
VrPHD11	EVM0015866	LG04	1446	214761	482
VrPHD12	EVM0020560	LG04	651	815261	217
VrPHD13	EVM0001604	LG04	1821	3550301	607
VrPHD14	EVM0010532	LG04	768	4296095	256
VrPHD15	EVM0018513	LG04	765	4303419	255
VrPHD16	EVM0017282	LG04	4275	10042863	1425
VrPHD17	EVM0009677	LG04	1878	11655184	626
VrPHD18	EVM0002357	LG04	744	40914641	248
VrPHD19	EVM0028597	LG04	2142	41606944	714
VrPHD20	EVM0023136	LG05	756	4282900	252
VrPHD21	EVM0013188	LG05	2208	9306327	736
VrPHD22	EVM0019892	LG05	1854	26151828	618
VrPHD23	EVM0019116	LG05	3084	31333422	1028
VrPHD24	EVM0029168	LG05	2478	40784001	826
VrPHD25	EVM0007886	LG05	1680	41667828	560
VrPHD26	EVM0020961	LG06	4092	13681543	1364
VrPHD27	EVM0002284	LG06	1695	19816251	565
VrPHD28	EVM0025253	LG07	2157	36418795	719
VrPHD29	EVM0014963	LG08	957	4048888	319
VrPHD30	EVM0028983	LG08	651	5849015	217
VrPHD31	EVM0004968	LG08	651	6197157	217
VrPHD32	EVM0025849	LG08	5199	29180330	1733
VrPHD33	EVM0032842	LG08	2628	29405319	876
VrPHD34	EVM0030091	LG08	6288	34751294	2096
VrPHD35	EVM0007906	LG08	3579	35654603	1193
VrPHD36	EVM0018531	LG08	2124	36147895	708
VrPHD37	EVM0016362	LG09	720	10305037	240
VrPHD38	EVM0028859	LG09	762	32156548	254
VrPHD39	EVM0004891	LG10	3846	694623	1282
VrPHD40	EVM0029631	LG10	3852	701776	1284

VrPHD41	EVM0021296	LG10	1938	17857410	646
VrPHD42	EVM0019470	LG10	735	27087034	245
VrPHD43	EVM0032256	LG10	2499	28255385	833
VrPHD44	EVM0003836	LG11	3945	8746300	1315
VrPHD45	EVM0016633	LG11	654	9035906	218
VrPHD46	EVM0019367	LG11	4857	21444457	1619
VrPHD47	EVM0005488	LG11	753	24171016	251

Supplementary Table S4. VrMYB gene family members and the physical length of their encoded proteins.

Gene name	Sequenced gene ID from 'Sulv No. 1'	Chromosome	Coding sequence length/bp	5′ upstream regions
VrMYB1	EVM0005560	Contig00179	861	11439
VrMYB2	EVM0010930	LG01	423	2206209
VrMYB3	EVM0028907	LG01	921	3243046
VrMYB4	EVM0020402	LG01	738	4602563
VrMYB5	EVM0021788	LG01	1086	6054516
VrMYB6	EVM0013881	LG01	939	10349067
VrMYB7	EVM0028481	LG01	570	11397937
VrMYB8	EVM0033126	LG01	1344	11524106
VrMYB9	EVM0008505	LG01	1110	11833301
VrMYB10	EVM0013440	LG01	816	13028333
VrMYB11	EVM0031927	LG01	915	15613695
VrMYB12	EVM0000792	LG01	867	16621775
VrMYB13	EVM0002267	LG01	678	24983609
VrMYB14	EVM0031317	LG01	1467	28545742
VrMYB15	EVM0032627	LG01	1353	30209517
VrMYB16	EVM0009300	LG01	1098	39796146
VrMYB17	EVM0022110	LG01	849	68027512
VrMYB18	EVM0010078	LG01	1539	69488081
VrMYB19	EVM0011624	LG01	897	70559509
VrMYB20	EVM0012999	LG01	945	71537640
VrMYB21	EVM0020709	LG02	966	3142603
VrMYB22	EVM0012396	LG02	1320	3869799
VrMYB23	EVM0017972	LG02	1215	4088298
VrMYB24	EVM0027599	LG02	1098	4417318
VrMYB25	EVM0014054	LG02	831	4429906
VrMYB26	EVM0011121	LG02	1062	4738854
VrMYB27	EVM0005254	LG02	1005	7464185
VrMYB28	EVM0031370	LG02	867	9305300
VrMYB29	EVM0033288	LG02	1011	15093994
VrMYB30	EVM0004411	LG02	1062	15246300
VrMYB31	EVM0029632	LG02	1188	20676906
VrMYB32	EVM0021718	LG02	609	20758026
VrMYB33	EVM0023869	LG02	600	20827528
VrMYB34	EVM0020010	LG02	1683	29622812
VrMYB35	EVM0007447	LG02	1065	29732215
VrMYB36	EVM0010411	LG02	672	33650729
VrMYB37	EVM0031136	LG02	927	46395122
VrMYB38	EVM0004125	LG02	954	49527657
VrMYB39	EVM0001237	LG02	1182	49898886

VrMYB40	EVM0007149	LG02	699	49925239
VrMYB41	EVM0000379	LG02	801	50745400
VrMYB42	EVM0027998	LG03	918	2310822
VrMYB43	EVM0000308	LG03	957	5693138
VrMYB44	EVM0013966	LG03	822	8523751
VrMYB45	EVM0032669	LG03	786	8536146
VrMYB46	EVM0014746	LG03	804	10100486
VrMYB47	EVM0030033	LG03	789	11121364
VrMYB48	EVM0000740	LG03	1188	14963087
VrMYB49	EVM0024395	LG03	825	25422649
VrMYB50	EVM0002797	LG03	951	26243106
VrMYB51	EVM0006103	LG03	864	39973532
VrMYB52	EVM0009478	LG03	777	47334208
VrMYB53	EVM0003357	LG03	1218	47821275
VrMYB54	EVM0032563	LG04	777	396247
VrMYB55	EVM0015313	LG04	951	2788234
VrMYB56	EVM0014326	LG04	768	3271492
VrMYB57	EVM0012150	LG04	1026	3830506
VrMYB58	EVM0001810	LG04	2937	6953904
VrMYB59	EVM0022926	LG04	999	9150779
VrMYB60	EVM0014112	LG04	1011	12166730
VrMYB61	EVM0032156	LG04	807	24384510
VrMYB62	EVM0023431	LG04	966	27350400
VrMYB63	EVM0000878	LG04	1104	32217610
VrMYB64	EVM0026777	LG04	1329	33226586
VrMYB65	EVM0025104	LG04	840	34725633
VrMYB66	EVM0006567	LG04	1182	35397298
VrMYB67	EVM0032849	LG04	1071	35817673
VrMYB68	EVM0000801	LG05	588	1728975
VrMYB69	EVM0026195	LG05	825	2521264
VrMYB70	EVM0030911	LG05	1107	3296488
VrMYB71	EVM0017989	LG05	936	3441646
VrMYB72	EVM0001430	LG05	2958	8083259
VrMYB73	EVM0027250	LG05	720	9005658
VrMYB74	EVM0032698	LG05	1248	9905352
VrMYB75	EVM0033888	LG05	969	30266155
VrMYB76	EVM0032706	LG05	969	35194041
VrMYB77	EVM0029407	LG05	903	35533286
VrMYB78	EVM0011065	LG05	714	38929320
VrMYB79	EVM0007833	LG06	1125	1916353
VrMYB80	EVM0029575	LG06	1008	7942042
VrMYB81	EVM0014377	LG06	840	8488661
VrMYB82	EVM0015956	LG06	669	9388041
VrMYB83	EVM0033641	LG06	1239	14323026

VrMYB84	EVM0016947	LG06	978	15175322
VrMYB85	EVM0011529	LG06	1545	18478480
VrMYB86	EVM0019160	LG06	1581	33195345
VrMYB87	EVM0000410	LG07	852	861694
VrMYB88	EVM0010808	LG07	1068	866950
VrMYB89	EVM0031365	LG07	699	970829
VrMYB90	EVM0019863	LG07	1200	2271094
VrMYB91	EVM0020569	LG07	960	2926494
VrMYB92	EVM0013532	LG07	1227	3194577
VrMYB93	EVM0003993	LG07	1071	5359807
VrMYB94	EVM0029404	LG07	1077	6342333
VrMYB95	EVM0012389	LG07	1137	6397663
VrMYB96	EVM0000108	LG07	726	7275559
VrMYB97	EVM0019692	LG07	960	13979286
VrMYB98	EVM0032776	LG07	2910	19758055
VrMYB99	EVM0032242	LG07	870	30791637
VrMYB100	EVM0019634	LG07	690	34421375
VrMYB101	EVM0003086	LG07	924	35991018
VrMYB102	EVM0000615	LG07	822	37594882
VrMYB103	EVM0015069	LG08	903	256269
VrMYB104	EVM0032717	LG08	642	266722
VrMYB105	EVM0028465	LG08	873	663218
VrMYB106	EVM0009222	LG08	963	15144821
VrMYB107	EVM0000815	LG08	1668	16541282
VrMYB108	EVM0011764	LG08	927	25920175
VrMYB109	EVM0002237	LG08	708	26022741
VrMYB110	EVM0033844	LG08	675	26072172
VrMYB111	EVM0003395	LG08	939	26085986
VrMYB112	EVM0018091	LG08	900	26104769
VrMYB113	EVM0014330	LG08	702	26171339
VrMYB114	EVM0020194	LG08	2991	29224878
VrMYB115	EVM0033351	LG08	927	29631740
VrMYB116	EVM0025016	LG08	780	31398411
VrMYB117	EVM0029784	LG08	984	32015910
VrMYB118	EVM0019797	LG08	765	33909838
VrMYB119	EVM0017526	LG08	1119	35157817
VrMYB120	EVM0016623	LG09	948	697288
VrMYB121	EVM0021429	LG09	1647	5868636
VrMYB122	EVM0024414	LG09	1059	6618162
VrMYB123	EVM0033800	LG09	1053	7063266
VrMYB124	EVM0030186	LG09	1080	7077548
VrMYB125	EVM0016640	LG09	885	27134757
VrMYB126	EVM0022694	LG09	870	30064211
VrMYB127	EVM0032009	LG09	879	31949320

VrMYB128	EVM0018698	LG10	912	924463
VrMYB129	EVM0017624	LG10	1488	1821853
VrMYB130	EVM0026842	LG10	540	2848375
VrMYB131	EVM0023558	LG10	1053	3639490
VrMYB132	EVM0011578	LG10	933	4204804
VrMYB133	EVM0000892	LG10	1002	9999840
VrMYB134	EVM0000585	LG10	1365	17279203
VrMYB135	EVM0019841	LG10	1422	19766638
VrMYB136	EVM0025775	LG10	861	22673020
VrMYB137	EVM0000554	LG10	1161	25241362
VrMYB138	EVM0029314	LG10	951	25321370
VrMYB139	EVM0008997	LG10	882	25357901
VrMYB140	EVM0002131	LG11	1533	1204593
VrMYB141	EVM0012904	LG11	1008	4768859
VrMYB142	EVM0029023	LG11	1071	6383608
VrMYB143	EVM0008154	LG11	1125	13333087
VrMYB144	EVM0025151	LG11	981	18867609
VrMYB145	EVM0026939	LG11	1323	19978885
VrMYB146	EVM0007962	LG11	999	21753314
VrMYB147	EVM0029299	LG11	738	22918933
VrMYB148	EVM0033679	LG11	939	23415204
VrMYB149	EVM0018474	LG11	768	24293515

Supplementary Table S5. Statistics analysis of stress responsive cis-acting elements of three gene families.

cis-acting element	VrWRKYs	VrPHDs	VrMYBs
cis-acting element involved in the abscisic acid responsiveness	72	32	128
auxin-responsive element	26	16	44
cis-acting element involved in defense and stress responsiveness	38	20	73
cis-acting element involved in gibberellin-responsiveness	9	10	20
cis-acting element involved in low-temperature responsiveness	25	16	50
cis-acting element involved in salicylic acid responsiveness	35	22	68
cis-acting regulatory element involved in the MeJA-responsiveness	58	25	89
gibberellin-responsive element	34	21	70
MYB binding site involved in drought-inducibility	29	23	71
MYB binding site involved in flavonoid biosynthetic genes regulation	11	5	15

Supplementary Table S6. Ka, Ks and Ka/Ks statistics of VrPHDs.

Gene ID	Gene ID	Duplication Type	Ka	Ks	Ka/Ks	Selection pressure
EVM0000716.1	EVM0004891.1	Segmental duplication	0.608846	3.02763	0.201096	Purifying selection
EVM0010532.1	EVM0023136.1	Segmental duplication	0.106927	0.555334	0.192545	Purifying selection
EVM0009677.2	EVM0013188.1	Segmental duplication	0.305717	0.70953	0.430873	Purifying selection
EVM0003660.1	EVM0016362.1	Segmental duplication	0.044382	0.804688	0.055154	Purifying selection
EVM0019116.1	EVM0020961.1	Segmental duplication	0.375849	0.766535	0.490322	Purifying selection
EVM0025849.1	EVM0019367.1	Segmental duplication	0.268316	0.86321	0.310835	Purifying selection
EVM0032842.1	EVM0010247.1	Segmental duplication	0.298153	1.369855	0.217653	Purifying selection
EVM0028859.1	EVM0005488.1	Segmental duplication	0.063336	0.528531	0.119835	Purifying selection
EVM0002357.1	EVM0005488.1	Segmental duplication	0.139951	2.262468	0.061858	Purifying selection
EVM0010532.1	EVM0028859.1	Segmental duplication	0.148637	2.487734	0.059748	Purifying selection
EVM0025592.1	EVM0025253.2	Segmental duplication	0.107268	0.705948	0.151949	Purifying selection
EVM0008784.1	EVM0028597.2	Segmental duplication	0.230662	0.663261	0.34777	Purifying selection

Supplementary Table S7. Ka, Ks and Ka/Ks statistics of VrMYBs.

Gene ID	Gene ID	Duplication Type	Ka	Ks	Ka/Ks	Selection pressure
EVM0029632.1	EVM0000410.1	Segmental duplication	0.473112	1.588943	0.297753	Purifying selection
EVM0017526.1	EVM0018698.1	Segmental duplication	0.188668	0.659658	0.286009	Purifying selection
EVM0021718.1	EVM0031365.1	Segmental duplication	0.110692	0.954724	0.115941	Purifying selection
EVM0015313.1	EVM0000801.1	Segmental duplication	0.23498	1.36923	0.171615	Purifying selection
EVM0010078.1	EVM0017624.1	Segmental duplication	0.10958	0.984717	0.111281	Purifying selection
EVM0018047.1	EVM0007833.1	Segmental duplication	0.233164	0.900907	0.25881	Purifying selection
EVM0012999.1	EVM0027998.1	Segmental duplication	0.405612	1.965135	0.206404	Purifying selection
EVM0014326.1	EVM0026195.1	Segmental duplication	0.260973	1.853106	0.14083	Purifying selection
EVM0000792.1	EVM0026195.1	Segmental duplication	0.298965	1.796247	0.166439	Purifying selection
EVM0008280.1	EVM0026842.1	Segmental duplication	0.218404	1.297695	0.168301	Purifying selection
EVM0013881.1	EVM0020569.1	Segmental duplication	0.34802	1.745868	0.199339	Purifying selection
EVM0032849.1	EVM0020569.1	Segmental duplication	0.28608	3.246829	0.088111	Purifying selection
EVM0004125.1	EVM0020569.1	Segmental duplication	0.167623	0.687218	0.243915	Purifying selection
EVM0000792.1	EVM0014326.1	Segmental duplication	0.328926	1.538982	0.213729	Purifying selection
EVM0012150.1	EVM0030911.1	Segmental duplication	0.18405	1.161935	0.1584	Purifying selection
EVM0031927.1	EVM0023558.1	Segmental duplication	0.224436	1.139604	0.196942	Purifying selection
EVM0031927.1	EVM0012150.1	Segmental duplication	0.38448	2.327404	0.165197	Purifying selection
EVM0014326.1	EVM0011578.1	Segmental duplication	0.35251	3.912017	0.09011	Purifying selection
EVM0000792.1	EVM0011578.1	Segmental duplication	0.137835	1.486039	0.092754	Purifying selection
EVM0028907.1	EVM0027599.1	Segmental duplication	0.370011	2.11733	0.174753	Purifying selection
EVM0028907.1	EVM0029575.1	Segmental duplication	0.167006	1.303629	0.128109	Purifying selection
EVM0001810.1	EVM0001430.1	Segmental duplication	0.211376	0.695283	0.304014	Purifying selection

EVM0009478.1	EVM0015956.2	Segmental duplication	0.385117	1.478664	0.260449	Purifying selection
EVM0011764.1	EVM0008154.1	Segmental duplication	0.386846	2.265264	0.170773	Purifying selection
EVM0033888.1	EVM0033641.1	Segmental duplication	0.376612	1.349445	0.279087	Purifying selection
EVM0012396.1	EVM0000740.1	Segmental duplication	0.159797	0.840361	0.190152	Purifying selection
EVM0012396.1	EVM0017795.1	Segmental duplication	0.407153	2.302842	0.176805	Purifying selection
EVM0017972.1	EVM0017795.1	Segmental duplication	0.144604	0.709872	0.203704	Purifying selection
EVM0027599.1	EVM0024350.2	Segmental duplication	0.241591	0.95713	0.252412	Purifying selection
EVM0011121.1	EVM0022532.1	Segmental duplication	0.353888	1.405667	0.251758	Purifying selection
EVM0032627.1	EVM0000585.1	Segmental duplication	0.240577	0.711082	0.338325	Purifying selection
EVM0007833.1	EVM0025151.1	Segmental duplication	0.301272	2.65182	0.113609	Purifying selection
EVM0011764.1	EVM0025151.1	Segmental duplication	0.199954	1.121547	0.178284	Purifying selection
EVM0003395.1	EVM0010422.1	Segmental duplication	0.348096	1.604702	0.216922	Purifying selection
EVM0031317.1	EVM0019841.1	Segmental duplication	0.162585	0.520139	0.31258	Purifying selection
EVM0005254.1	EVM0007962.1	Segmental duplication	0.304141	1.418899	0.21435	Purifying selection
EVM0033351.1	EVM0007962.1	Segmental duplication	0.169585	0.623753	0.271879	Purifying selection
EVM0025016.1	EVM0029299.1	Segmental duplication	0.181655	0.779206	0.233129	Purifying selection
EVM0029784.1	EVM0033679.1	Segmental duplication	0.16186	0.679031	0.238369	Purifying selection
EVM0013532.1	EVM0000554.1	Segmental duplication	0.377977	2.076523	0.182024	Purifying selection
EVM0022339.1	EVM0000554.1	Segmental duplication	0.229561	1.38011	0.166335	Purifying selection
EVM0001237.1	EVM0000554.1	Segmental duplication	0.364029	2.680058	0.135829	Purifying selection
EVM0031370.1	EVM0024395.1	Segmental duplication	0.376433	1.524352	0.246946	Purifying selection
EVM0018047.1	EVM0011764.1	Segmental duplication	0.339362	4.305054	0.078829	Purifying selection
EVM0005254.1	EVM0002797.1	Segmental duplication	0.214366	0.823959	0.260166	Purifying selection
EVM0010519.1	EVM0026347.1	Segmental duplication	0.062654	0.706862	0.088637	Purifying selection
EVM0031136.1	EVM0016640.1	Segmental duplication	0.137599	0.63609	0.21632	Purifying selection
EVM0022110.1	EVM0023431.1	Segmental duplication	0.346596	1.600597	0.216542	Purifying selection
EVM0005254.1	EVM0033351.1	Segmental duplication	0.34188	1.337482	0.255614	Purifying selection
EVM0014377.1	EVM0022694.1	Segmental duplication	0.17198	1.162155	0.147984	Purifying selection
EVM0020402.1	EVM0032009.1	Segmental duplication	0.403001	1.631901	0.246952	Purifying selection
EVM0028465.1	EVM0029784.1	Segmental duplication	0.380262	2.262362	0.168082	Purifying selection
EVM0008505.1	EVM0000878.2	Segmental duplication	0.126094	0.813598	0.154983	Purifying selection
EVM0033126.1	EVM0026777.1	Segmental duplication	0.273565	0.950373	0.28785	Purifying selection
EVM0032563.1	EVM0019797.1	Segmental duplication	0.188179	0.743449	0.253116	Purifying selection
EVM0020709.1	EVM0032706.1	Segmental duplication	0.190711	0.754974	0.252606	Purifying selection
EVM0013881.1	EVM0032849.1	Segmental duplication	0.187579	0.97417	0.192553	Purifying selection
EVM0004125.1	EVM0032849.1	Segmental duplication	0.279223	2.671849	0.104505	Purifying selection
EVM0012999.1	EVM0003086.1	Segmental duplication	0.254087	0.557411	0.455835	Purifying selection
EVM0011624.1	EVM0000615.1	Segmental duplication	0.253692	0.966077	0.262601	Purifying selection
EVM0020402.1	EVM0006002.1	Segmental duplication	0.32773	1.829381	0.179148	Purifying selection
EVM0030033.1	EVM0009478.1	Segmental duplication	0.130525	0.844633	0.154535	Purifying selection
EVM0013881.1	EVM0004125.1	Segmental duplication	0.351883	2.124027	0.165668	Purifying selection
EVM0022339.1	EVM0001237.1	Segmental duplication	0.43457	2.469325	0.175987	Purifying selection
